# Role of microRNAs in obesity and obesity-related diseases

**DOI:** 10.1186/s12263-017-0577-z

**Published:** 2017-09-25

**Authors:** Giuseppe Iacomino, Alfonso Siani

**Affiliations:** Institute of Food Sciences, CNR, Via Roma, 64, 83100 Avellino, Italy

**Keywords:** Obesity, Metabolic disease, Disease biomarkers, miRNAs, microRNA

## Abstract

In recent years, the link between regulatory microRNAs (miRNAs) and diseases has been the object of intensive research. miRNAs have emerged as key mediators of metabolic processes, playing crucial roles in maintaining/altering physiological processes, including energy balance and metabolic homeostasis. Altered miRNAs expression has been reported in association with obesity, both in animal and human studies. Dysregulation of miRNAs may affect the status and functions of different tissues and organs, including the adipose tissue, pancreas, liver, and muscle, possibly contributing to metabolic abnormalities associated with obesity and obesity-related diseases. More recently, the discovery of circulating miRNAs easily detectable in plasma and other body fluids has emphasized their potential as both endocrine signaling molecules and disease indicators. In this review, the status of current research on the role of miRNAs in obesity and related metabolic abnormalities is summarized and discussed.

## Background

### Obesity: a global epidemic

About 10 years ago, the World Health Organization indicated the increasing prevalence of overweight and/or obesity worldwide as a challenge for public health, due the adverse consequences associated with obesity and overweight [1, 2]. The trend has been so steep and sudden that some researchers refer to it as an “epidemic.” Nowadays, over 60% of the United States (USA) adult population is considered overweight or obese, but the high prevalence of obesity is not limited to the USA, being observed in industrialized as well as in least developed countries. Even more worrisome is the rapidly increasing prevalence of obesity among children observed over the last 30 years [3]. Epidemiological studies have established a firm association between an elevated BMI and chronic conditions such as diabetes, dyslipidemia, hypertension, heart disease, non-alcoholic fatty liver disease, and some types of cancer [4, 5]. Dyslipidemia and type 2 diabetes (T2D) have exhibited a corresponding increase over the same time span also in children [6].

The medical costs of obesity, and also the growing mortality among obese individuals, are likely related to comorbid conditions rather than obesity per se. A surplus in energy intake and a limited physical activity are considered among the driving factors of obesity; however, the contribution of genetic and epigenetic traits could not be disregarded. In the last decade, large and well-powered studies have shown that multiple loci on the human genome are associated with obesity and obesity-related phenotypes [7–9].

### A new layer of control

The individual susceptibility to weight gain and the associated clinical effects may largely vary due to differences in the genetic background, lifestyle, and environmental stimuli. It is well recognized that the “common” obesity is the result of the interplay of environmental factors with genetic factors reflecting the additive contribution of many genes that confer different degrees of susceptibility (polygenic obesity) [10]. Of note, most of the genes associated to obesity predisposition are also related to food intake and regulation of energy balance [11], with about 20–40% of the variance in energy and macronutrient intake explained by genetic effects [12]. Recent studies suggest that as much as 21% of BMI variation can be explained by common genetic variants [13]. Even though the genetics of obesity has been extensively explored, most of the genetic variability in BMI remains unexplained and, in addition, the confirmation of the effects of single candidate genes or their combination is still incomplete.

Genomes contain information that is mandatory to build and run cells, including the self-coordination responsible to define complex organs and ultimately to self-assemble an organism by driving cellular differentiation and morphogenesis programs. Together, these processes require contribution of information-dense and dynamic regulatory systems involving a number of mechanisms including transcription factors, DNA methylation, ATP-dependent chromatin remodeling mechanisms, and post-translational modifications of histones, as well the dynamic acetylation and deacetylation of core histones [14–16]. Virtually, any step of the gene expression flow is finely controlled, and the discovery of small non-coding RNAs (ncRNAs) has added new critical players to the wide range of existing mechanisms [17].

**Fig. 1:**
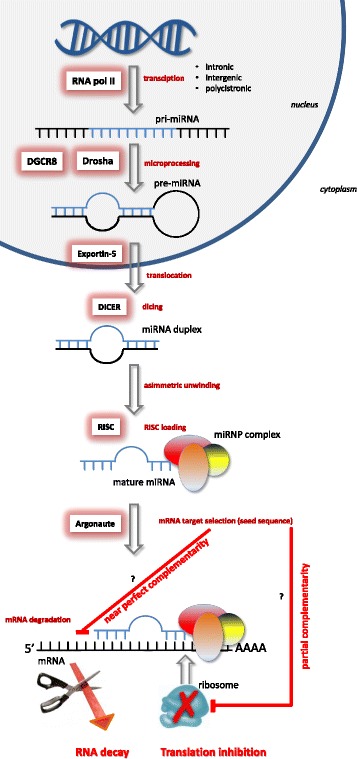
miRNA biogenesis. Single miRNAs are transcribed by RNA polymerase II (pri-miRNA) from genomic DNA. The pri-miRNA is processed to pre-miRNA by Drosha-DGCR8. The pre-miRNA is transferred to the cytoplasm by exportin-5 where it is recognized and cleaved by the DICER complex to create a miRNA duplex. The duplex unwinds, and the mature miRNA assembles into RISC. The miRNA base-pairs with target mRNA determines gene silencing via mRNA cleavage or translation repression depending on the degree of homology between the miRNA “seed” to the 3′ UTR target sequence of the mRNA

In a few years, microRNA (miRNA) research has proceeded from the discovery of a non-coding RNA in *C. elegans* [18, 19] to thousands of publications describing their critical connection to a variety of cell processes and diseases [20]. miRNAs are short ncRNAs, with a length of 20–24 nucleotides, which are engaged in the control of gene expression programs [21–23]. At present, more than 2000 different miRNAs have been described in humans, and their number is still increasing in the miRBase database [24]. The release of 21 of the repository contains 28,645 entries representing hairpin precursor miRNAs, expressing 35,828 mature miRNA products, in 223 species. In recent years, miRNA biogenesis and mechanisms of action have been thoroughly described as illustrated in Fig. 1 [25–31]. miRNAs are essential elements of the cell epigenetic machinery which post-transcriptionally repress the expression of target genes, usually by binding to the 3′ UTR of messenger RNA, contributing to the regulation of many biological processes [32].

For base-pairing with a target mRNA, nucleotides in positions 2–8 of a miRNA are relevant. This sequence motif is referred to as “seed sequence” and is closely connected to the recognition of a mRNA target. However, other regions also contribute in determining the target specificity [33]. A numeric designation has been given in sequential order to individual miRNAs according to the discovery date, and matching miRNAs found in different organisms have been assigned through the same numeric code with a species-specific preface. Moreover, miRNAs have been clustered into families constructed on seed region similarity, which is accountable for the corresponding ability to target common groups of gene transcripts [34]. Although some miRNAs exhibit a tissue-specific localization, most miRNAs show a broader tissue distribution [35]. Each miRNA can simultaneously regulate large cohorts of transcripts, and individual mRNA may include multiple binding sites for different miRNAs originating an intricate regulatory network [32, 36]. Even though miRNAs usually act as slight modulators, defining only a weak inhibitory effect on a single target, more often, they coordinately affect multiple transcripts in a signaling pathway or nodes correlated in complex regulatory networks, exercising significant cumulative effects. A notable example is given by the members of the miR-200 family, acting at multiple levels as enforcers of the epithelial phenotype. Actually, they target both cytoskeletal effectors, regulating actin filament organization, and pathways that locally coordinate the cytoskeleton organization to preserve cell morphology and prevent cell migration [37].

Computational and experimental analyses support the view that endogenous miRNAs may comprehensively influence the expression of up to 60% of mouse and human genes [38, 39] and that a huge number of miRNAs are under the control of relevant signal transduction cascades. Therefore, miRNAs have been reported to be involved in a countless cellular processes, including proliferation, differentiation, DNA repair, apoptosis, and metabolism [40, 41]. Additionally, increasing evidence indicates that miRNA dysregulation is causative and/or indicative of several diseases, including cancer [42–45]. Substantial progress has been made in interpreting the role of individual miRNAs in a number of biological settings. As an example, members of the highly conserved miR-34 family act as tumor suppressor. Dysregulation or loss of the host gene from which this miRNA is derived is associated with cancer progression in numerous cell types [45].

## miRNA detection

miRNA profiling is a key step which requires sensitive and reproducible detection methods. A number of different techniques have been developed to determine miRNAs in biological samples, such as NGS (RNAseq), reverse transcription quantitative PCR, and microarray, each method having its own strengths and weaknesses [46]. In general, miRNA characterization, as compared to mRNA profiling techniques, is more difficult to perform because procedures should be able to discriminate miRNAs differing by as little as a single nucleotide, also taking into account differences between mature miRNAs and their precursors (which also encompass the sequence of the mature miRNA species).

Furthermore, precise measurement of circulating miRNAs can be challenging, due to their relatively low concentration, to the presence of undesired inhibitors potentially interfering in the downstream quantification procedures and, finally, to confounding sources of intracellular miRNAs that may contaminate the extraction process. Indeed, the inconsistencies and dissimilar results reported among different studies could be partially explained by differences in both detection procedures and experimental setup. The source of miRNAs, the extraction procedures, the quantities used in profiling analysis workflow, and the methods of data analysis all together possibly contribute to the uncertainty still observed in the literature, highlighting the need for reproducible and well-standardized methods [47–49].

## miRNAs in obesity and metabolic diseases

At the time of our search, 61,363 published papers concerning miRNAs were found on PubMed. Most of them deal with human diseases/disorders and a growing number of reports about miRNAs as useful clinical tools [50], in particular with regard to the identification of “circulating” miRNAs (see the “Circulating miRNAs” section) as cancer biomarkers [51, 52]. Omics studies have indeed demonstrated that changes in miRNA profiles of various tissues (e.g., pancreas, adipose tissue, and liver) correlate with obesity [8] and several metabolic diseases [53, 54]. There are intriguing reports suggesting that miRNAs may be regulated by diet and lifestyle factors [55] and could be responsive to various nutritional interventions [56].

For the purpose of the present review, we thoroughly explored PubMed using different combinations of the subsequent keywords: “microRNA,” “circulating miRNAs,” “adipose tissue,” “adipogenesis,” “obesity,” “diabetes,” and “metabolic diseases.” Obviously, a complete and comprehensive scrutiny of the available literature was outside our scope. Actually, the most cited research papers as well as the most recent and complete reviews on this research area were included.

### miRNAs in the adipose tissue

The obesity–diabetes connection has been long time established, having its roots in interdependent alterations of glucose and lipid metabolism. Adipose tissue, the storage site of triglycerides, is the key machinery where energy homeostasis is regulated, to the extent that adipose tissue is now considered an endocrine organ (see Table 1) [57, 58]. In this context, it is not surprising that miRNAs may contribute to the regulation of energy balance and metabolic homeostasis, by controlling a wide range of metabolic pathways [54].

**Table 1:** White adipose tissue in brief

White adipose tissue achieves metabolic functions through the release of signaling molecules, such as adipokines, and hundreds of diverse factors, including classical hormones such as leptin, growth factors such as IGF-1 and PDGF, and cytokines such as IL-6, IL-8, or TNF-α acting as inflammatory mediators [163], comprehensively linked to appetite regulation and insulin sensitivity. Growing evidence supports the notion that chronic low-grade inflammation is a basic characteristic of obesity contributing to the establishment of insulin resistance into the target organs, including the adipose tissue, liver, and muscle, and the vascular system [164]. Excess of nutrients, such as lipids and glucose, may simultaneously trigger inflammatory responses, which further disrupt metabolic function, enhancing stress and inflammation. Accordingly, the nutrition–immunity theory suggests that in the adipose tissue, overnutrition-induced obesity triggers low-level inflammatory processes [165]. Anomalous fat accumulation has been shown to increase pathogenic risks since adipose tissue remains in a state of subclinical chronic inflammation [166]. This condition is strictly related to a massive recruitment of macrophages and to an increased immune cell proliferation activation–infiltration connected to adipocyte hypertrophy and an impaired adipogenesis [167]. The latter process is tightly controlled by a mixture of regulatory signals including endocellular transcription factors, extracellular circulating hormones [168], and additional post-transcriptional regulators of gene expression [60, 169–172]. Adipogenesis is the process during which fibroblast-like pre-adipocytes differentiate into mature adipocytes, a complex mechanism involving cell commitment, clonal expansion, and terminal differentiation [173]. More in detail, differentiation from pre-adipocytes into mature adipocytes is a key step orchestrated by several transcription factors such as the peroxisome proliferator-activated receptor-γ (PPARγ) and CCAAT/enhancer-binding proteins (C/EBPs) that coordinately control expression of genes required for adipocyte phenotypes. C/EBPβ and C/EBPδ are induced by adipogenic stimuli and represent primary regulators of adipogenesis. Targets of C/EBPβ and C/EBPδ are the promoters of the genes encoding critical adipogenic factors such as C/EBPα and PPARγ, as well as the sterol regulatory element-binding protein (SREBP1), the key regulator of lipogenic genes. The protein encoded by the C/EBPα intronless gene is a leucine zipper transcription factor which can bind to specific promoters and enhancers as a homodimer or it can form heterodimers with the related proteins C/EBPβ and C/EBPγ. The C/EBPα protein has been shown to bind to the promoter and to also modify the expression of the leptin gene, playing a central role in body weight homeostasis. C/EBPα is sufficient to trigger differentiation of pre-adipocytes into mature adipocytes [174]. Peculiarly, PPARγ directly triggers endogenous C/EBPα transcription. In turn, C/EBPα activates the PPARγ gene through a positive feedback loop, thereby promoting adipogenesis [168]. Together, PPARγ and C/EBPα promote the expression of genes involved in insulin sensitivity, in lipogenesis and lipolysis, and, ultimately, in the terminal differentiation and mature functions of adipocytes. The complex series of events governing adipose cell commitment and differentiation also includes anti-adipogenic signaling cascade controlled by Wnt, BMPs, TGF-β, and hedgehog [175]. As expected, an increase in size (hypertrophy) and number (hyperplasia) of adipocytes causes higher level of fat mass and energy storage in the adipose tissue, possibly ending in increased obesity risk.	

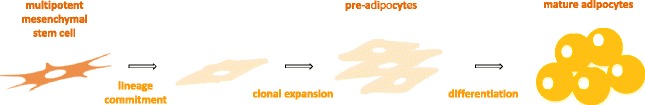

The first evidence suggesting a role of miRNAs in fat cells regulation was in *Drosophila*, showing that miR-14 exerts a suppressive effect on fat metabolism by targeting p38 and MAPK [59]. Subsequentely, a wide array of miRNAs involved in the regulation of glucose and lipid metabolism was identified, with particular focus on adipocyte differentiation, control of β-cell mass, and insulin signaling pathway in both physiological and pathological conditions [60, 61]. However, the information regarding the possible mechanisms is still limited [62]. As an example, the miRNAs reported in Table 2 have been shown to possibly promote adipogenesis through different mechanisms, while other species (Table 3) have been reported to interfere with adipocyte differentiation [63].

**Table 2:** Adipogenesis promoting miRNAs

miRNA	Target/process	Reference
miR-17	Rb2/p130	[176]
miR-21	TGF-β signaling pathway	[88]
miR-26b	Adipogenic differentiation by inhibiting PTEN. Levels are reduced in the visceral adipose tissue	[81, 177]
miR-30	Modulation of RUNX2, a key regulator of osteogenesis	[178]
miR-103	MEF2D. AKT/mTOR signal pathway	[179]
miR-143	MAPK signaling pathway. Glucose homeostasis	[180]
miR-146b	SIRT1-induced deacetylation of FOXO1	[181]
miR-148a	Wnt signaling pathway	[182]
miR-181	TNF-α	[183]
miR-199a	Smad1	[184]
miR-204	Runx2	[185]
miR-210	Wnt signaling pathway	[65]
miR-320	RUNX2	[186]
miR-371	Epigenetic modifications. Adipogenic differentiation	[187]
miR-375	ERK1/2 signaling pathway	[77]
miR-378	Adipocyte development and differentiation	[188]
miR-637	Sp7	[189]

**Table 3:** Anti-adipogenic miRNAs

miRNA	Target/process	Reference
let-7	FABP4 and PPARγ signaling pathway. Antiregulates clonal expansion via HMGA2	[74]
miR-15a	Delta-like 1 homolog	[190]
miR-22	HDAC6	[191]
miR-27a/b	MAPK/ERK signaling via PHB, C/EBPβ, PPARγ, and aP2 signaling. Attenuates lipid accumulation	[65]
miR-31	C/EBPα signaling.	[192]
miR-33b	Decrease lipogenesis via early B cell factor 1 (EBF1) targeting C/EBPα and PPARγ signaling	[193]
miR-93	Sirt7 and Tbx3	[194]
miR-125a	ERRα	[195]
miR-130	Inhibition of adipogenesis by inhibiting PPARγ	[66]
miR-138	Inhibition of adipocyte differentiation via EID-1. Lipid droplet reduction	[196]
miR-145	Preadipocyte differentiation by targeting IRS1	[197]
miR-155	C/EBPβ pathway	[198]
mirR-193a/b	Adiponectin production in the adipose tissue. Induce myoblast differentiation into brown adipocytes	[199]
miR-194	Stimulates osteogenesis and inhibits adipogenesis via COUP-TFII	[200]
miR-221	Adiponectin signaling	[198]
miR-222	Glucose metabolism. Altered by insulin administration	[198]
miR-224	EGR2. Fatty acid metabolism	[201]
miR-344	GSK3β, activating the Wnt/β-catenin signaling pathway. Decreases triglyceride accumulation	[65]
miR-363	Inhibits adipocyte differentiation by targeting E2F3. Downregulates C/EBPα and PPARγ	[73]
miR-365	Brown fat differentiation	[202]
miR-369	FABP4. Adipogenic differentiation	[187]
miR-448	KLF5. Activation of serotonin receptors 5-HT2AR and 5-HT2CR	[203]
miR-709	GSK3ß of Wnt/ß-catenin signaling	[204]

miR-143 and miR-130 are the best studied among the miRNAs linked to adipogenesis. miR-143 and miR-145 are often investigated together, since they are closely located and can be co-transcribed. miR-143 has been identified as a positive regulator of human adipocyte differentiation acting via ERK5 signaling. Expression of miR-143 and miR-145 is upregulated in the liver of mouse models of obesity, and the iperexpression of miR-143 impairs insulin-stimulated AKT activation and glucose homeostasis. On the contrary, mice lacking for the miR-143–145 cluster did not develop the obesity-associated insulin resistance [64]. Another notable example is given by miR-27a and miR-130a that inhibit adipocyte differentiation through PPARγ downregulation [65, 66]. The overexpression of miR-27a and miR-130a clearly suppresses adipocyte differentiation along with PPARγ expression. Lower expression levels of miR-130a and miR-130b have been reported in the abdominal subcutaneous adipose tissue and in the plasma of obese women compared with those of lean subjects [67]. In contrast, circulating miR-130b has been found to be higher in obese children [68]. An interesting study from Wang et al. identified miR-130b as a potential biomarker for overweight, hypertriacylglycerolemia, and metabolic syndrome, suggesting a mechanism linking obesity and obesity-related metabolic diseases, through an adipose–muscle crosstalk mediated by circulating miRNAs [69]. They have also found that the addition of TGF-β in matured 3T3-L1 adipocytes dramatically elevated the level of miR-130b in the culture medium, while slightly decreasing intracellular level of miR-130b, thus confirming that this miRNA is released from differentiating adipocytes during adipogenesis. Other miRNAs affect lineage determination. As an example, miR-124 has a pro-adipogenic effect by targeting Dlx5, a pro-osteogenic transcription factor that determines cell fate in human bone marrow-derived mesenchymal stem cells [70].

miRNAs can be expressed from separate transcripts or from a primary transcript structured in co-transcribed clusters encoding more than one miRNA (polycistronic) [71]. The best-characterized polycistronic miRNA cluster is represented by miR-17-92, encoding for miR-17, miR-18a, miR-19a, miR-20a, miR-19b-1, and miR-92a [72]. This cluster is overexpressed during adipocyte clonal expansion and acts by directly repressing the RB family Rb2/p130, so controlling the RB-E2F-mediated checkpoint. In the same pathway, miR-363 inhibits adipocyte differentiation by targeting E2F and concomitantly downregulating C/EBPα and PPARγ [73].

Let-7 was the first human miRNA discovered. This miRNA is included in a well-conserved family counting 11 members associated with many critical cell functions (e.g., apoptosis, proliferation, and cell cycle checkpoints). This miRNA family directly regulates oncogenes such as RAS and HMGA2 and plays a significant role in developmental processes. Moreover, miRNAs of this family regulate glucose metabolism and peripheral insulin resistance by targeting IGF1R, insulin receptor (INSR), and insulin receptor substrate-2. Let-7 negatively controls adipogenesis by regulating the expression of high-mobility group AT-hook2. Let-7 is upregulated in the model of 3T3-L1 adipogenesis. The ectopic introduction of let-7 in 3T3-L1 and 3T3-F442A cells decreased the clonal expansion as well as the terminal differentiation [74]. Overall, there is an indication that let-7 acts as an anti-adipogenic factor controlling the transition from clonal expansion to terminal differentiation. Moreover, let-7 has been shown to be directly involved in glucose metabolism and insulin resistance acting on targets associated with the insulin/IGF-1R pathway in mice [75]. In let-7 knockout mice, animals with a reduced expression of let-7 did not develop insulin resistance despite diet-induced obesity, suggesting that let-7 may represent an interesting therapeutic target for diabetes [76].

Various miRNAs affect adipocyte differentiation by targeting C/EBPs and insulin signaling. miR-375 has been shown to promote 3T3-L1 adipocyte differentiation by increasing mRNA levels of C/EBPα and PPARγ2 and by inducting adipocyte fatty acid-binding protein (aP2) and triglyceride accumulation. Conversely, miR-375 suppresses phosphorylation levels of ERK1/2 in 3T3-L1 cells [77].

There is evidence that miR-206 plays a key role in the growth and development of the skeletal muscle, promoting the myogenic differentiation and has been related to the pathogenesis of numerous diseases, including heart failure, chronic obstructive pulmonary disease, Alzheimer’s disease, and some cancers [78]. In most of these diseases, miR-206 is downregulated, suggesting this miRNA as a “disease-avoiding” molecule [78]. Interestingly, miR-206 expression is abundant in brown adipocytes in mice but missing in white adipocytes [79]. Moreover, miR-206 suppresses liver X receptor α (LXRα), a gene target of PPAR, thus inhibiting lipogenesis and controlling lipid metabolism in HepG2 cells [80]. Another miRNA involved in the regulation of adipogenic differentiation is miR-26b [81]. Overexpression of miR-26b in 3T3-L1 cells significantly accelerated the mRNA expression of adipogenic markers, PPARγ, fatty acid synthase (FAS), C/EBPα, and lipoprotein lipase, and increased lipid accumulation, by inhibiting the PTEN expression. In contrast, inhibition of miR-26b expression decreased cell differentiation [81].

Current findings indicate that miR-146b expression in 3T3-L1 is evidently increased during adipogenesis [82]. Sirtuin 1 (SIRT1) is negatively regulated by miR-146b. SIRT1 promotes gene transcription by deacetylating various transcription factors, including the forkhead box O1 (FOXO1). The role of SIRT1 as a regulator of metabolic homeostasis has been extensively investigated. SIRT1 level is decreased during adipogenesis. SIRT1, by interacting with the PPARγ co-repressors N-CoR and SMRT, inhibits PPARγ and prevents adipogenesis. Accordingly, differentiation of 3T3-L1 cells is induced by overexpression of miR-146b, and on the contrary, inhibition of miR-146b reduces adipocyte differentiation in 3T3-L1 [83].

The highly conserved miR-8/miR-200 family consists of a single ortholog in the fruit fly (miR-8) and of five members in the vertebrates (miR-200a, miR-200b, miR-200c, miR-141, and miR-429) [84]. miR-8/miR-200 have been reported as repressors of the evolutionarily conserved Wnt/wingless pathway in the *Drosophila* eye and in mouse mesenchymal stem cells, controlling the eye size and the differentiation of the mesenchymal stem cells into adipocytes, respectively [85]. Drosophila miR-8 and human miR-200 family also prevent the expression of an inhibitor of the insulin/phosphoinositide-3 kinase (PI3K) signaling in fat body and liver cells, thus controlling fat body/liver cell growth and proliferation [86]. In particular, overexpression of members of this miRNA family increases adipogenesis, the level of fatty acid-binding protein 4 (FABP4), and lipid accumulation.

Liang et al. showed that the expression of miR-210 was highly increased during 3T3-L1 adipogenesis. Transfection of miR-210 mimics into 3T3-L1 cells promoted the expression of adipogenic markers and adipocyte differentiation by targeting SHIP1, a negative regulator of the PI3K/Akt pathway. Additionally, ectopic inhibition of the endogenous miR-210 during adipogenesis possibly blocks adipocyte differentiation [87].

Likewise, miR-21 in 3T3-L1 cells significantly promotes adipocyte differentiation and increases adiponectin expression, while decreasing AP-1 protein level. miR-21 may enhance differentiation of human adipose-derived stem cells by direct inhibition of the TGF-β receptor 2 expression [88].

Current evidence indicates that inflammation induces a specific miRNA response in adipocytes with effects on the physiopathology of obesity-induced inflammation of adipose tissue [89]. As an additional example, a research in mice identified a pro-inflammatory loop mediated by NF-κB and miR-155 that could participate in the amplification of inflammatory status in adipocytes [90].

An interesting paper from Thomou et al. has recently defined a new role for adipose tissue and its potential implications in the mechanism of cell crosstalk [91]. The authors have established the role of adipose tissue as a major source of circulating miRNAs, which can regulate gene expression in distant tissues so acting as regulators of metabolism. Mice with an adipose tissue-specific knockout of Dicer miRNA-processing enzyme, as well as humans with lipodystrophy, display an extensive decline in the levels of circulating miRNAs. Transplantation of both white and brown adipose tissues reestablishes the level of many circulating miRNAs associated with an improvement in glucose tolerance and a reduction in hepatic fibroblast growth factor 21 (FGF21) mRNA and circulating protein. FGF21 plays a critical role in metabolism, stimulating the fatty acid oxidation in the liver and glucose uptake in fat. Of note, levels of FGF21 are significantly increased in patients with T2D and non-alcoholic fatty liver disease and positively correlate with BMI in humans, indicating obesity as a possible FGF21-resistant state [91].

### miRNAs in the pancreas

The endocrine pancreas plays a major role in regulating glucose homeostasis through the secretion of insulin and glucagon. Alterations of pancreatic hormone production and activity are causally linked to diabetes. T2D is a complex disease characterized by pancreatic islet dysfunction and insulin resistance in peripheral tissues. Declined insulin levels in T2D have been attributed to a decrease in β-cell function/mass [92]. Identity and dedifferentiation of β-cells may also contribute to the insulin production decay. The first suggestion for a role of miRNAs in hormone secretion in vertebrates came from a cloning approach of small RNAs from the β-cell-derived line MIN6 [93]. Comparing islet–cell miRNA profiles with those of 15 other human tissues, a panel of 40 miRNAs predominantly expressed in the islets have been recently identified [94]. Numerous miRNAs have been reported to be involved in pancreatic development, with some of them playing positive roles, while others exhibiting negative effects [95, 96]. One of the most relevant is miR-375, which is the most abundant in pancreatic islets and is essential in maintaining normal pancreatic β-cell mass [97]. An increase in miR-375 expression is observed during pancreatic islet cell development, whereas β-cell functioning is linked to its decrease [98]. Numerous genes associated with cellular growth are controlled by this miRNA during human pancreas development [99]. Moreover, miR-375 targets a number of transcription factors, such as PDX1, HNF6, and INSM1, engaged in pancreatic islet functioning [100]. Interestingly, the transcription factor neurogenin3 (Ngn3), considered as an early marker of pancreatic islet cells with a prominent role during the development of the endocrine lineages in mice [101], also interferes with miR-375 expression. Additional miRNAs, such as miR-15a, miR-15b, miR-16, and miRNA-195, also target Ngn3. Remarkably, miR-375 has been reported to be involved in the modulation of insulin secretion in stimulated cell line MIN6 [93]. More in detail, miR-375 leads to a reduced glucose-stimulated insulin secretion by downregulating myotrophin mRNA (encoding a key protein involved in cell membrane fusion with insulin granules) and therefore inhibiting exocytosis. Furthermore, it has been shown that miR-375 concurrently downregulates expression of insulin by targeting the phosphoinositide-dependent kinase-1 in INS1-E cells [102]. Other miRNAs such as miR-7 and miR-124 have been recognized to be engaged in regulation of β-cell differentiation and establishment of pancreatic islets [97]. High levels of miR-7 are detectable in the pancreatic cells, both in the developing and adult phases [103]. Overexpression of miR-7 in pancreatic progenitors has been shown to impair the differentiation of both α- and β-cells and is associated with a repression of Pax6 expression. The knockdown of miR-7 during early embryonic life determines an overall downregulation of insulin production, a decrease in the number of β-cells, and the onset of glucose intolerance in the postnatal period. Furthermore, an in vitro inhibition of miR-7 promotes death of β-cell in explanted pancreatic buds. In summary, data suggest that dysregulation of miR-7 signaling network in response to metabolic stress or cellular insults contribute to the loss of β-cell identity and establishment of T2D [104].

Other miRNAs, like miR-146a and miR-34a, seem overexpressed only during the differentiation processes and have been shown to contribute, at least partially, to cytokine-mediated β-cell dysfunction occurring during the initial phases of type 1 diabetes in non-obese diabetic (NOD) mice [54]. Further, miRNAs expressed in pancreatic islets, such as miR-143 and let-7, have been connected to glucose homeostasis by targeting key insulin signaling components [75].

Other pancreatic functions can be modulated by miRNAs. For instance, miR-29, in addition to its ability to regulate β-cell proliferation, has also been shown to negatively regulate insulin secretion by directly targeting *Stx-1a* involved in insulin exocytosis [105]. Similarly, miR-124a, miR-9, and miR-96 can regulate insulin release by β-cells [106]. During the late pancreas development, miR-124a is upregulated [107]. This miRNA targets mRNA of both cAMP-responsive element-binding protein 1 (Creb1) and forkhead box protein A2 (Foxa2). Notably, Foxa2 modulates the expression of the insulin gene in multiple pathways responsible for the secretion of this hormone, mainly through an upstream regulation of pancreatic and duodenal homeobox 1 (Pdx1). Pdx1 is critical for glucose balance and pancreas development and together with Ngn3 is required for β-cell differentiation. Moreover, miR124a increases the levels of SNAP25, Rab3A, and synapsin-1A and decreases those of Rab27A and Noc2, targets involved in the exocytotic mechanisms for insulin release [106].

Overexpression of miR-9 in insulin-secreting INS-1E cells results in a reduction of insulin exocytosis. mir-9 acts by downregulating the expression of the transcription factor Onecut-2 and, in turn, by increasing the level of Granuphilin/Slp4, a Rab GTPase effector associated with β-cell secretory granules [108].

Finally, miR-29 also controls insulin secretion by regulating the monocarboxylate transporter 1 (Mct1) expression [105].

### miRNAs in the muscle

The skeletal muscle represents the major user of glucose in human body, accounting for about 75% of insulin-mediated glucose uptake. Several miRNAs, referred to as myomiR family, are preferentially detectable in muscle tissue and act as modulators of skeletal and cardiac muscle myogenesis, proliferation, and metabolism, as well as hypertrophy. The myomiRs include miR-1, miR-133a, miR-133b, miR-206, miR-208a, miR-208b, miR-486, and miR-499 [109]. miR-206 is specifically expressed in the skeletal muscle, whereas miR-208a is cardio-specific; nevertheless, most of these miRNAs are co-expressed in the heart and skeletal muscles [110]. MyomiRs have been proven to directly target pathways regulating skeletal muscle homeostasis; their deregulation is observed across cardiac and muscular dysfunctions [111]. As an example, a reduced expression of miR-133 is observed in mouse and human models of cardiac hypertrophy, with several studies connecting this miRNA to the pathogenesis of heart diseases [112]. Interestingly, it has been proven that acute exercise determines an increase in the levels of miR-1, miR-133a, and miR-206 [113], important molecules possibly driving cell-to-cell communication. A recent paper from Zhou et al. has demonstrated the involvement of miR-29a in the induction of insulin resistance by targeting PPARδ in rats’ skeletal muscle cells. Overexpression of miR-29a in the cell line C2C12 suppresses the expression of PPARδ, finally affecting the expression of its coactivator PGC-1α. PPARδ/PGC-1α-dependent signaling determines a decrease in levels of glucose transporter 4, the principal glucose transporter in the skeletal muscle, which partially induces a decrease in insulin-dependent glucose uptake and adenosine triphosphate (ATP) availability [114]. Similarly, another study found that miR-29a levels are elevated in the diabetic (db/db) mouse liver and its overexpression prevents insulin-mediated inhibition of hepatic phosphoenolpyruvate carboxykinase (PEPCK) gene expression, which is normally implicated in inhibition of gluconeogenesis and suppressed in diabetes [115]. Other studies have shown that high-fat diet significantly increases the expression of miR-29a in myocytes, impairing insulin signaling and glucose uptake through an extensive decrease in insulin receptor substrate 1 (IRS-1). Possibly, the upregulation of miR-29a by saturated fatty acids (SFA) is causally related to the development of insulin resistance in the muscle [116]. miR-106b, highly expressed in the muscle of diabetic subjects, has been associated to skeletal muscle insulin resistance and T2D. Overexpression of miR-106b determines mitochondrial dysfunction and insulin resistance in C2C12 myotubes by targeting mitofusin-2. Notably, expression of this miRNA is improved following TNF-α treatment, suggesting that its enhanced production under chronic low-grade inflammation may represent a valuable link between mitochondrial alteration and T2D [117].

A fascinating research topic is the pleiotropic regulatory network exerted by miR-208a, a heart-specific miRNA that also controls glucose metabolism and energy homeostasis. The heart contributes to regulate systemic energy homeostasis via MED13 [118], a subunit of the Mediator complex, which governs the transcription by the thyroid hormone (that enhances energy expenditure and regulates body weight) and other nuclear hormone receptors [119]. MED13 is negatively controlled by miR-208a. Remarkably, anti-miR-208 oligonucleotides confer resistance to diet-induced obesity and improve glucose tolerance in mice [120].

### miRNAs in the liver

miRNAs control various functions in the liver, and cumulative evidence suggests that they have a relevant role in this organ pathology [121]. miR-122 is a dominant hepatocyte-specific miRNA accounting for about 75% of total miRNA expression in human hepatocytes with approximately 135,000 copies, making it one of the highly expressed in the human body. Levels of miR-122 are controlled by liver-enriched transcription factors (LETFs), including hepatocyte nuclear factor (HNF) 6 and 4a. Interestingly, miR-122 regulatory network has been implicated in numerous liver functions, ranging from cholesterol metabolism, stress responses, viral infection, cancer, and circadian regulation of hepatic genes [122]. The role of this miRNA is also emerging in the metabolic syndrome and other liver diseases, such as liver inflammation related to alcohol use, autoimmune processes, and the development of liver fibrosis both in human and animal models. Pathological suppression of miR-122 has been described in hepatocellular carcinoma [123], non-alcoholic steatohepatitis [124], and liver cirrhosis [121]. This miRNA is intensely investigated because of its role in cholesterol metabolism. Antisense inhibition of miR-122 in normal mice results in lower levels of serum cholesterol, LDL, and serum triglyceride and increased hepatic fatty acid oxidation. These effects on lipid metabolism have been associated with the expression of key genes involved in fatty acid metabolism and cholesterol biosynthesis, including the rate-limiting enzyme 3-hydroxy-3-methylglutaryl-CoA-reductase [125]. Similarly, antisense inhibition of this miRNA in chimpanzee provokes a plasma cholesterol reduction supporting its key role in maintaining liver homeostasis [126]. Since miR-122 can be detected in blood, it has been proposed as a circulating biomarker of liver injury in chronic hepatitis B and C, non-alcoholic fatty liver disease, and drug-induced liver disease [127].

Other miRNAs, such as miR-27b, miR-33, miR-34, miR-103, miR-104, 223, and miR-370, also control the fatty acid metabolism and cholesterol biosynthesis in the liver. As an example, miR-27b could exert regulatory effects in the lipid metabolism and is altered in dyslipidemia, theoretically affecting both liver and heart functions in mouse [128]. Moreover, miR-34a targets hepatic SIRT1. The upregulation of miR-34a, with a concomitant decrease in SIRT1 levels, has been described in fatty livers of mice with diet-induced obesity [129]. Additionally, the mitochondrial enzyme carnitine palmitoyl transferase, involved in the transport of long-chain fatty acids across the membrane, is targeted by miR-370 that concurrently affects lipid metabolism [130].

MiR-33-3p regulates cholesterol and lipid metabolism as well as fatty acid oxidation [131]. This miRNA downregulates several genes encoding key enzymes involved in fatty acid metabolism, cholesterol efflux, such as ATP-binding cassette A1 (ABCA1), and insulin signaling. This miRNA in vitro targets IRS2 and SIRT6 genes involved in insulin signaling. Inhibition of miR-33 in non-human primates resulted in elevated plasma HDL and protective effects against atherosclerosis. However, recent studies suggest that miR-33 inhibition may have adverse effects on lipid and insulin metabolism in mice [132].

Hepatic miR-223 has been shown to reduce cholesterol biosynthesis in mice by targeting the 3-hydroxy-3-methylglutaryl-CoA synthase 1 and the sterol-C4-methyloxidase-like protein. Moreover, this miRNA inhibits the HDL-C uptake by targeting the scavenger receptor class B member 1 and promotes cholesterol efflux by positively regulating the expression of ABCA1. Notably, miR-223 level is controlled by the cholesterol levels [133].

miR-26a additionally regulates insulin signaling as well as metabolism of glucose and lipids in mice and humans [134]. Overweight compared with lean subjects exhibit a decreased liver expression of miR-26a. Overexpression of this miRNA in mice fed a high-fat diet enhanced insulin sensitivity and reduced hepatic glucose and fatty acid synthesis, so preventing obesity-induced metabolic complications [134].

Remarkably, a number of hepatic miRNAs have been reported to be dysregulated in obese patients with NAFLD and NASH [124, 135, 136].

### Circulating miRNAs

Although miRNAs were first identified inside cells, more recently, an increasing number of miRNAs have been found, in surprisingly high concentrations, in plasma and other body fluids such as serum, urine, and saliva [137]. The concept that miRNAs could be stable in blood and body fluids [138], in spite of the ubiquity of nucleases, was originally met with skepticism by the scientific community. However, this characteristic generated high interest for the possibility that variations in cell-free miRNA expression could be used as non-invasive biomarkers for several diseases and, possibly, as early diagnostic tools. [139]. Due to their accessibility, the most common miRNA sources investigated are whole blood, serum, and plasma [140].

Circulating miRNAs (cmiRNAs), as expected, are not naked molecules, and two major mechanisms have been identified to shield them from nuclease activity. The first one consists in the formation of complexes of specific binding proteins, such as Argonaute 2 (AGO-2) [141], a protein involved in the RNA silencing complex, with high-density lipoproteins [142], or nucleophosmin-1 (NPM-1), a nucleolar RNA-binding protein implicated in the nuclear export of the ribosome [143]. The second proposed mechanism stems from the discovery of cmiRNAs enclosed within circulating microvesicles or exosomes [144] deriving either from the endosomal compartments or from the cell plasma membrane [145]. Although an established mechanism for the release of miRNAs from cells remains largely unknown, growing evidence supports the indication that extracellular miRNAs, arranged either into exosomes or protein complexes, may be delivered to the receiver cells, where they can be engaged in the control of target gene translation [146]. However, the physiological role of circulating miRNAs is still uncertain.

Differential cmiRNA profiles have been reported in individuals with obesity and T2D [147]. In Table 4, the behavior of specific cmiRNAs in different metabolic disorders is summarized. For instance, miR-126 is reduced in T2D [148] and has been proposed as a biomarker of endothelial dysfunction caused by uncontrolled glycemia in T2D [149]; miR-1, miR-21, miR-133a, and miR-208 are enriched in the plasma after myocardial infarction [150]; miR-122 is enhanced in hepatic injury and steatosis [151], as well as let-7e in hypertension [152]. Additionally, circulating miR-130a and miR-195 have been connected with high blood pressure [153]. Alterations in circulating miR-23a, miR-27a, miR-130, miR-195, miR-197, miR-320a, and miR-509-5p have been associated to metabolic syndrome [153, 154]. Moreover, cmiRNA profiles exhibited a sex-specific association with metabolic syndrome [155]. Circulating let-7b, miR-143, and 221 have been proposed to regulate atherogenic and adipogenic processes [156]. Furthermore, the expression of circulating miR-17-5p and miR-132 was decreased in obesity, mirroring the expression pattern of miRNAs in omental fat from the same group of obese subjects [157]. Different cmRNA profiles have also been described in pre-gestational and gestational obesity [158].

**Table 4:** Summary of the current knowledge on circulating miRNAs in the context of obesity and metabolic diseases

miRNA	Process	Reference
↓miR-126	T2D	[148]
↑miR-1, miR-21, miR-133a, and miR-208	Myocardial infarction	[150]
↑miR-122	Hepatic injury and steatosis	[151]
↑let-7e	Hypertension	[152]
↑miR-130a and miR-195	Blood pressure	[153]
miR-23a, miR-27a, miR-130, miR-195, miR-197, miR-320a, and miR-509-5p	Metabolic syndrome	[153, 154]
↑let-7g and miR-221	Female-specific in metabolic syndrome	[155]
let-7b, miR-143, and miR-221	Atherogenic as and adipogenic processes	[156]
↓miR-17-5p and miR-132	Obesity	[157]
↓miR-122, miR-324-3p, miR-375, and miR-652; ↑miR-625	Pre-gestational and gestational obesity	
↑miR-140-5p, miR-142-3p, miR-222; ↓miR-532-5p, miR-125b, miR-130b, miR-221, miR-15a, miR-423-5p, and miR-520c-3p	Morbidly obese patients	[67]
↓miR-17-5p	Coronary artery disease	[205]
↑miR-33	Familial hypercholesterolemia and cardiometabolic disorders	[206]
↑miR-122	NAFLD. Insulin resistance, obesity, metabolic syndrome, type 2 diabetes, and an adverse lipid profile	[207, 208]
↑miR-122 and miR-199a	Children obesity	[161]
↓miR-375	T1D onset	[209]

Ortega et al. have reported that morbidly obese patients exhibit a marked increase of circulating miR-140-5p, miR-142-3p, and miR-222 and a decrease of miR-532-5p, miR-125b, miR-130b, miR-221, miR-15a, miR-423-5p, and miR-520c-3p. In the same study, a surgery-induced weight loss caused a significant decrease of circulating miR-140-5p, miR-122, miR-193a-5p, and miR-16-1 and an increase of miR-221 and miR-199a-3p [67].

Furthermore, various studies have shown a differential cmiRNA signature in overweight/obese as compared in normal weight children and adolescents [68, 159–161], thus suggesting that these molecules could have a promising role in the early identification of children at risk of excess body fat accumulation and related metabolic abnormalities.

## Conclusion

**Fig. 2:**
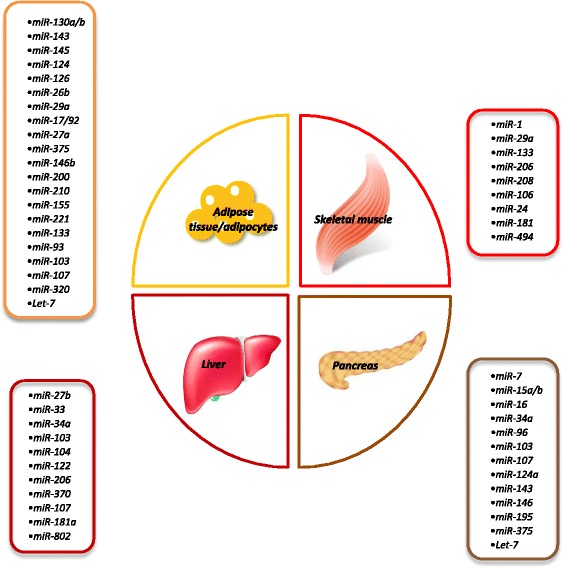
Overview of miRNAs possibly associated with obesity and metabolic diseases in different tissues. Circulating miRNAs are additionally reported in Table 4

Since their first detection in 1993, miRNAs have attracted growing interest among the scientific community. Considerable progress has been achieved in the research of contributory crosstalk between regulatory miRNAs and diseases. miRNAs have emerged as key regulators of lipid and glucose metabolism and play pivotal roles in the onset of obesity and obesity-related diseases by affecting status and functions of the adipose tissue, pancreas, liver, and muscle (Fig. 2). However, information about the mechanisms of action remains nearly limited, due to the miRNAs’ ability to simultaneously affect multiple pathways/gene networks and to the technical limitations of in vivo profiling [48].

A comprehensive understanding of the role of miRNAs in tissue metabolism and energy homeostasis may in perspective open the road to therapeutics strategies. Two main approaches are currently considered: the inhibition strategy, which uses anti-miR sequences able to target a specific miRNA and block its function, and the replacement therapy employing miRNA mimics [162].

The exciting emergence of circulating miRNAs as stable and accessible molecules opened a promising research avenue for the detection of non-invasive biomarkers potentially useful to the early identification of subjects at risk of excess body fat accumulation and related metabolic abnormalities.

For the etiological characterization, prospectively designed studies are strongly needed. A number of miRNA candidate signatures have been defined, and clinical trials are ongoing to validate their significance.

## Abbreviations

ABCA1: ATP-binding cassette A1
AGO: Argonaute
BMI: Body mass index
C/EBPs: CCAAT/enhancer-binding proteins
cmiRNAs: circulating miRNAs
Creb1: cAMP-responsive element-binding protein 1
ERK: Extracellular signal-regulated kinases
FABP4: Fatty acid-binding protein 4
FAS: Fatty acid synthase
FGF21: Fibroblast growth factor 21
Foxa2: Forkhead box protein A2
FOXO1: Forkhead box O1
HNF: Hepatocyte nuclear factor
INSR: Insulin receptor
IRS-1: Insulin receptor substrate 1
LETFs: Liver-enriched transcription factors
*LXRα*: Liver X receptor α
Mct1: Monocarboxylate transporter
miRNAs: microRNAs
NAFLD: Non-alcoholic fatty liver disease
NASH: Non-alcoholic steatohepatitis
N-CoR: Nuclear receptor corepressor
ncRNAs: Small non-coding RNAs
Ngn3: neurogenin3
NGS: Next-generation sequencing
NOD: Non-obese diabetic mice
NPM-1: Nucleophosmin-1
Pdx1: Pancreatic and duodenal homeobox 1
PEPCK: Phosphoenolpyruvate carboxykinase
PI3K: Insulin/phosphoinositide-3 kinase
PPARγ: Proliferator-activated receptor-γ
RB: Retinoblastoma susceptibility protein
RISCs: RNA-induced silencing complexes
RNAseq: RNA sequencing
SFA: Saturated fatty acids
SHIP1: SH2 (Src homology 2)-containing inositol phosphatase-1
SIRT1: Sirtuin 1
SMRT: Silencing mediator for retinoid and thyroid hormone receptors
SREBP1: Sterol regulatory element-binding protein
T2D: Type 2 diabetes
